# Computational Modeling of PI3K/AKT and MAPK Signaling Pathways in Melanoma Cancer

**DOI:** 10.1371/journal.pone.0152104

**Published:** 2016-03-25

**Authors:** Francesco Pappalardo, Giulia Russo, Saverio Candido, Marzio Pennisi, Salvatore Cavalieri, Santo Motta, James A. McCubrey, Ferdinando Nicoletti, Massimo Libra

**Affiliations:** 1 Department of Drug Sciences, University of Catania, 95125, Catania, Italy; 2 Department of Biomedical and Biotechnological Sciences, University of Catania, 95125, Catania, Italy; 3 Department of Mathematics and Computer Science, University of Catania, 95125, Catania, Italy; 4 Department of Engineering, University of Catania, 95125, Catania, Italy; 5 Department of Microbiology and Immunology, Brody School of Medicine at East Carolina University, Greenville, NC, United States of America; Rutgers University, UNITED STATES

## Abstract

**Background:**

Malignant melanoma is an aggressive tumor of the skin and seems to be resistant to current therapeutic approaches. Melanocytic transformation is thought to occur by sequential accumulation of genetic and molecular alterations able to activate the Ras/Raf/MEK/ERK (MAPK) and/or the PI3K/AKT (AKT) signalling pathways. Specifically, mutations of B-RAF activate MAPK pathway resulting in cell cycle progression and apoptosis prevention. According to these findings, MAPK and AKT pathways may represent promising therapeutic targets for an otherwise devastating disease.

**Result:**

Here we show a computational model able to simulate the main biochemical and metabolic interactions in the PI3K/AKT and MAPK pathways potentially involved in melanoma development. Overall, this computational approach may accelerate the drug discovery process and encourages the identification of novel pathway activators with consequent development of novel antioncogenic compounds to overcome tumor cell resistance to conventional therapeutic agents. The source code of the various versions of the model are available as S1 Archive.

## Introduction

RAS/RAF/MEK/ERK and PI3K/AKT/mTOR pathways represent fundamental signaling transduction and regulatory networks for the majority of cellular physiological processes, such as proliferation, differentiation and cell survival.

These pathways are mostly activated by alterations in Ras, B-RAF, PI3K, and PTEN genes [1]. Activation of such pathways is responsible of uncontrolled cell proliferation and can contribute to drug resistance. Combination therapies with pharmacological inhibitors of these pathways may have potential uses for the suppression of cancer cell proliferation and in turn may be efficacy to revert resistance. Malignant melanoma is a good tumor model to investigate the activation of RAS/RAF/MEK/ERK and the PI3K/AKT/mTOR pathways as it is frequently affected by B-RAF^V600E^ mutation that causes the activation of MAPK pathway [2]. It is an aggressive tumor of the skin with a poor prognosis for patients with advanced disease and it seems to be resistant to current therapeutic approaches.

Melanocytic transformation is thought to occur by sequential accumulation of genetic and molecular alterations [3, 4]. Although, the pathogenic mechanisms underlying melanoma development are still largely unknown, several genes and metabolic pathways have been shown to carry molecular alterations in melanoma. Melanomas exhibit mutations in the RAS/RAF/mitogen activated protein kinase (MAPK) pathway. It has been shown that 50% cutaneous melanomas exhibit B-RAF^V600E^ mutations, which results in an amino acid substitution at position 600 in B-RAF, from a valine (V) to a glutamic acid (E). This mutation is known to play a key role in proliferation and survival of melanoma cells, through activation of the MAPK pathway [5]. In particular, it occurs within the activation segment of the kinase domain and it results in an increased activity of the kinase itself. Constitutive activation of the kinase activity leads to unresponsitivity of negative feedback mechanisms within the MAPK pathway [6].

Additionally, an interaction between the MAPK and the phosphatidylinositide 3-kinase (PI3K)/AKT pathways has been found in cutaneous melanoma [7]. Interestingly, these studies suggest that MAPK and AKT pathways are activated in parallel and the evidence that PI3K/AKT and MAPK/ERK1/2 cascades are interconnected is largely described [8, 9, 10]. There are multiple cross-talk points between these two pathways, whose coordinated action determines the cell fate [11]. It is not surprising that the PI3K/AKT and MAPK pathways influence each other at different stages of signal propagation, both negatively and positively, resulting in dynamic and complex cross-talk. According to these findings, MAPK and AKT pathways could represent promising therapeutic targets for an otherwise devastating disease.

Computer simulations and computational modeling are useful to analyze and to increase knowledge of metabolic pathways and their complex interactions with the aim to understand the mechanisms of resistance to conventional drug therapy in melanoma [12, 13, 14].

In this work we develop a computational model that simulates both PI3K/AKT and MAPK pathways and their interactions, in order to analyze the cascade reactions responsible for melanoma development. Moreover, we modeled the behavior of the malignant melanoma A375 cell line, harboring B-RAF^V600E^ mutation, under the treatment of Dabrafenib, a commercial selective B-RAF inhibitor, recently approved in the treatment of patients with BRAF V600E mutation-positive advanced melanoma [15].

Overall, this model may be used for an in silico lab to study the effects of potential inhibitors that may improve the response to standard treatments.

## Methods

### Computational model of MAPK and PI3K/AKT pathways

In order to understand the effects of B-RAF alterations on both RAF-ERK and PI3K-AKT pathways we started from the model developed by Brown and collaborators [16]. In their work, the authors presented a computational model of the Epidermal Growth Factor (EGF) and Nerve Growth Factor (NGF) activated ERK pathway in PC12 cells, containing 13 different protein species and 16 biochemical reactions. Our model was developed using COPASI (COmplex PAthway SImulator), a software for simulation and analysis of biochemical networks and their dynamics [17]. Our model has considerably expanded the Brown model. It consists of 48 species and 48 biochemical reactions.

To include all the entities and their respective interactions useful to the target of this study, we retrieved all the needed information from KEGG (Kyoto Encyclopedia of Genes and Genomes) PATHWAY Database [18]. In particular, we focused on the interactions between two specific pathways: MAPK signaling pathway (Kegg reference: ko04010) and PI3K-AKT signaling pathway (Kegg reference: hsa04151). We studied the complex behaviour of the most important protein kinase cascades that involve the epidermal growth factor receptor (EGFR), phosphatidylinositol-4,5-bisphosphate 3-kinase (PIK3CA), RAC serine/threonine-protein kinase (AKT) and RAF proto-oncogene serine/threonine-protein kinase (RAF1). This inevitably lead us to consider the other signalling pathways that showed a correlation with the ones that may induce Dabrafenib resistance phenomena. To this end we also included into the model Ras signalling pathway (Kegg reference: ko04014) and mTOR signalling pathway (Kegg reference: ko041150) to investigate any possible mechanisms not observed before.

Relative initial concentrations of entities included in our model were gathered from GSE22301 available on GEO (Gene Expression Omnibus) dataset (http://www.ncbi.nlm.nih.gov/geo/) including expression profiling of several melanoma cell line as well as A375 used in this study. Microarray matrix data were normalized by log transformation using MeV data analysis tool [19]. Summarizing, we used KEGG database to understand and model the pathway flow and the GEO dataset to get the A375 cell line expression profiling; moreover, we set the values of the concentrations of the proteins as found by Brown et al.

The laws that governed the activations/deactivations in the Brown model were based on Henri-Michaelis-Menten kinetics. This kinetic law is one of the most common model of enzyme kinetic. Its mathematical form is the following:

V*Substrate/Km+Substrate.

It describes the rate of enzymatic reactions, in which “V” represents the maximum rate reached by the system, while Henri-Michaelis-Menten constant (Km) is the substrate concentration at which the reaction rate is half of V [20].

We slightly modified the classical Henri-Michaelis-Menten law to take into account both the substrate and the modifier that plays a specific role when we considered reactions that activated (and/or deactivated) specific proteins. The equation of the modified Henri-Michaelis-Menten is:

Kcat*Substrate1*Substrate2/(Km+Substrate2).

Kcat represents the number of enzymatic reactions catalyzed per second and the equation includes two types of substrates. Substrate1 stands for the modifier of the reaction, while Substrate2 is the generic substrate. It results more suitable for our purposes because it analyses the ratio between the reactions of the system and their affinity for the substrate taking in account the efficiency of the modifier involved.

All the biochemical reactions used in our model can be therefore divided into four main classes: *i)* activation/deactivation reactions modeled with a modified Henri-Michaelis-Menten law (for example Raf1Inactive becomes activated (Raf1Active) through RasActive); *ii)* reactions that physiologically inactivate species, modeled with mass action law (for example C3G deactivation); *iii)* proteins degradation modeled with mass action law (for example Dabrafenib degradation); *iv)* proteins production modeled with irreversible constant flux law (for example, the production of free RTK).

One of the targets of the model was to analyze the dynamics of critical nodes in A375 melanoma cell line harboring B-RAF^V600E^ mutation. Therefore, we modeled this cell line as follows: *i)* we introduced the new species bRafMutated with the same initial concentration of bRafInactive of 120000 mmol/ml; *ii)* we deleted the B-RAF activation by Rap1 as the new species bRafMutated is not affected by this signalling (the same applies for Ras); *iii)* we inhibited the deactivation of B-RAF by Raf1PPtase (as Raf1PPtase does not anymore influence B-RAF); *iv)* bRafMutated substitutes the bRafActive species in triggering the Mek activation. The other target of the model is to use it as an in silico lab to analyze the behavior of specific therapeutic treatments against melanoma, in particular in its mechanisms of resistance, with the aim to suggest new strategies that can be used in these circumstances. We, therefore, inserted into the model the features to reproduce the effect of Dabrafenib inhibitor in the complex dynamics of the PI3K/AKT pathway. To do this task, we added the Dabrafenib species (at different concentrations, see Results section). Then we modeled two specific reactions i.e., the normal drug degradation and the main effect of Dabrafenib in the inhibition of the bRafMutated species. From the specific literature, it is reported that the half-life of Dabrafenib is 10 hours (European Medicines Agency web site: http://www.ema.europa.eu). We used this parameter to set the associated mass action law to reproduce its decay.

Another important aspect not considered in Brown’s model is that all the receptors are very rapidly triggered by EGF and consequently they remain constitutively activated because their model did not take into account any reaction of degradation of receptors. We modeled this aspect inserting a degradation process based on an irreversible mass action law affecting both the free and bound RTK receptors. Moreover, the original Brown EGF model did not include the C3G/Rap1 pathway, a fundamental key point for the activation of B-RAF and consequently on ERK dynamics. To this end we modeled the activation of the C3G species through bound RTK receptor, and the Rap1 activation through the activated C3G protein.

**Fig 1:**
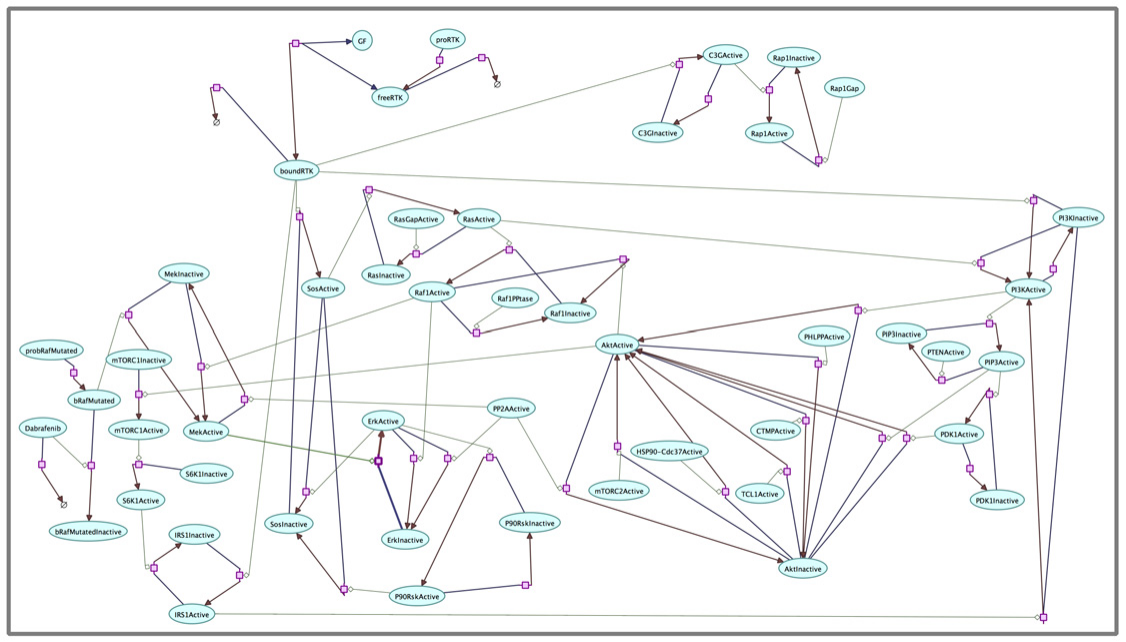
Diagram of PI3K-AKT and MAPK signaling pathways implemented into the model. Activation/deactivation reactions (modeled with a modified Henri-Michaelis-Menten law) are shown in the following way: the modifier i.e., the catalyst that triggers the reaction is depicted by a thin light green line ending with a rhombus; the involved species are connected by arrows starting with a blue color (input species) and ending with a brown color (resulting species). For example, Raf1Inactive becomes activated (Raf1Active) through RasActive. Reactions that physiologically inactivate species (modeled with mass action law) are depicted by an arrow starting with a blue color and ending with a brown color, for example C3G deactivation. Proteins degradation (modeled with mass action law) are depicted with the considered species connected by an arrow ending with an empty set symbol, for example Dabrafenib degradation. Proteins production (modeled with irreversible constant flux law) are depicted with the considered species connected by an arrow (for example, the production of free RTK). The Arcadia software (http://arcadiapathways.sourceforge.net) has been used to produce the graphical representation of the model.

**Fig 2:**
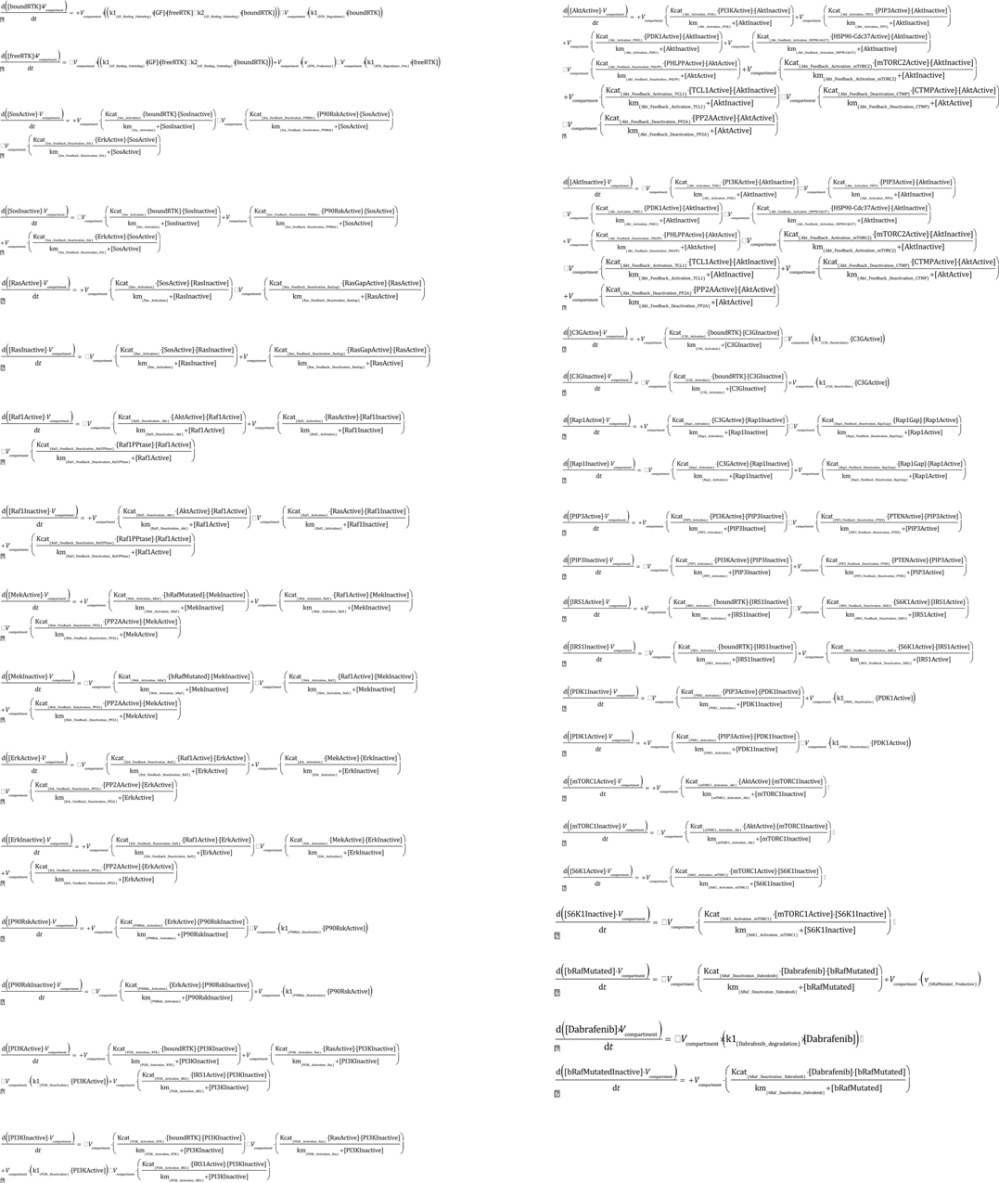
Complete list of the ordinary differential equations implemented in the model. The available versions of the Copasi models can be accessed as S1 Archive.

Furthermore, we deeply analyzed the fundamental role of AKT protein kinase in the crosstalk between the two main pathways involved. In particular, we focused on the role of AKT on the activation of mTORC1 pathway and on the activation/deactivation machinery of several proteins on AKT signaling. The resulting implementation of the pathways model, along with the relative set of ODEs can be found looking at the Figs 1 and 2.

### Cell line culture and treatment

A375 cell line was obtained from ATCC (LGC Standards, Italy). This cell line derived from a 54 year old female with malignant melanoma and represents a good model for studying the role of MAPK and AKT pathways because it is affected by the single alteration displayed in the B-RAF gene (V600E) (See Cosmic web site, http://cancer.sanger.ac.uk/cosmic). In fact, the presence of other genetic alterations, such as the mutations in KRAS or NRAS genes, may determine the activation of MAPK pathway.

The A375 melanoma cell line, obtained from ATCC (LGC Standards, Italy), were cultured in a humidified 5% CO2 incubator at 37°C with RPMI-1640 supplemented with 2 mmol/L L-glutamine, 100 IU penicillin, 100 μg/ml streptomycin and 10% fetal bovine serum (FBS), purchased from LONZA (Walkersville, USA). The A375 were plated in 70 mm cell-culture dishes at density of 500.000 cells and after 24h were treated for 48h with Dabrafenib (Selleck Chemical, USA) to final concentration of 2, 1, 0.5, 0.25 and 0.125 nM. Dimethyl Sulfoxide (DMSO) (Sigma-Aldrich, USA) was used as control.

### Western blot analysis

Protein profiling of treated A375 cell lines was analyzed by western blot using the Anti-MAP Kinase ERK1/ERK2 (pThr202/ pThr204) rabbit Ab (cat. n. 442685) and Anti-MAP Kinase ERK1/ERK2 rabbit Ab (cat. n. 442704) supplied from Merck Millipore (Darmstadt, Germany) to detect phosphorylated and total ERK 1/2 proteins, respectively. The Anti-beta Tubulin Ab (ab 15568- Abcam, Cambridge, UK) was used as housekeeping gene. Chromogenic detection of proteins was performed with Novex HRP Chromogenic (Invitrogen, USA). Western blot images were analyzed with image J software. All experiments were performed in triplicate. Student's t-test was used for statistical analysis.

## Results

**Fig 3:**
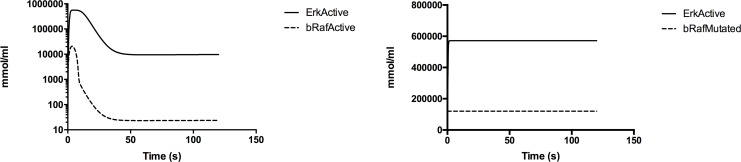
pERK (ErkActive) and B-RAF dynamics. Left panel shows the behavior under normal GF stimulation while right panel depicts the dynamics with B-RAF (bRafMutated) mutation in A375 cell lines.

We simulated our model under normal generic growth factor (GF) stimulation conditions to verify that it gave a strong transient activation of ERK. Then we simulated the A375 cell line with the B-RAF mutation. In this case, we expected elevated ERK activity that is characteristic of B-RAF^V600E^ mutated melanomas. Fig 3 shows the dynamics of both activated ERK (pERK) and B-RAF. Left panel highlights the normal condition case; while, right panel shows B-RAF mutated A375 cell line scenario. Finally, the simulation predicts correctly the expected behavior i.e., the species ErkActive has a constant elevated activity. Due to the nonlinearities of the presented model and to the high number of nodes and interactions inside the pathway, even if there is a clear relationship between bRafActive and ErkActive, we cannot say that there is a linear relationship between the two.

In particular, higher levels of ErkActive are not observed probably due to the fact that ErkActive is already near to its threshold levels and/or due to the contribution of other nodes in the pathway that may influence the final outcome.

Moreover, we simulated the behavior of the A375 cell lines under different concentrations of Dabrafenib inhibitor. B-RAF inhibition results in the limitation of pERK activity. Accordingly, in Table 1 the percentage of the inhibition of pERK is shown as result of pERK and ERK ratio. Concordant results were obtained analyzing in vitro and in silico data.

**Table 1:** pERK inhibition percentages in treated cell line.

Dabrafenib (nM)	In vitro inhibition (%)	In silico inhibition (%)
DMSO	100	100
0.125	84.3	93
0.250	60.1	64
0.500	38.1	36
1.0	19.2	19
2.0	16.7	9

Percentages of pERK inhibition are shown during different dosage treatment of B-RAF Dabrafenib inhibitor in A375 cell line. DMSO represents the control case. The ratio is normalized taking as reference the DMSO control values.

**Fig 4:**
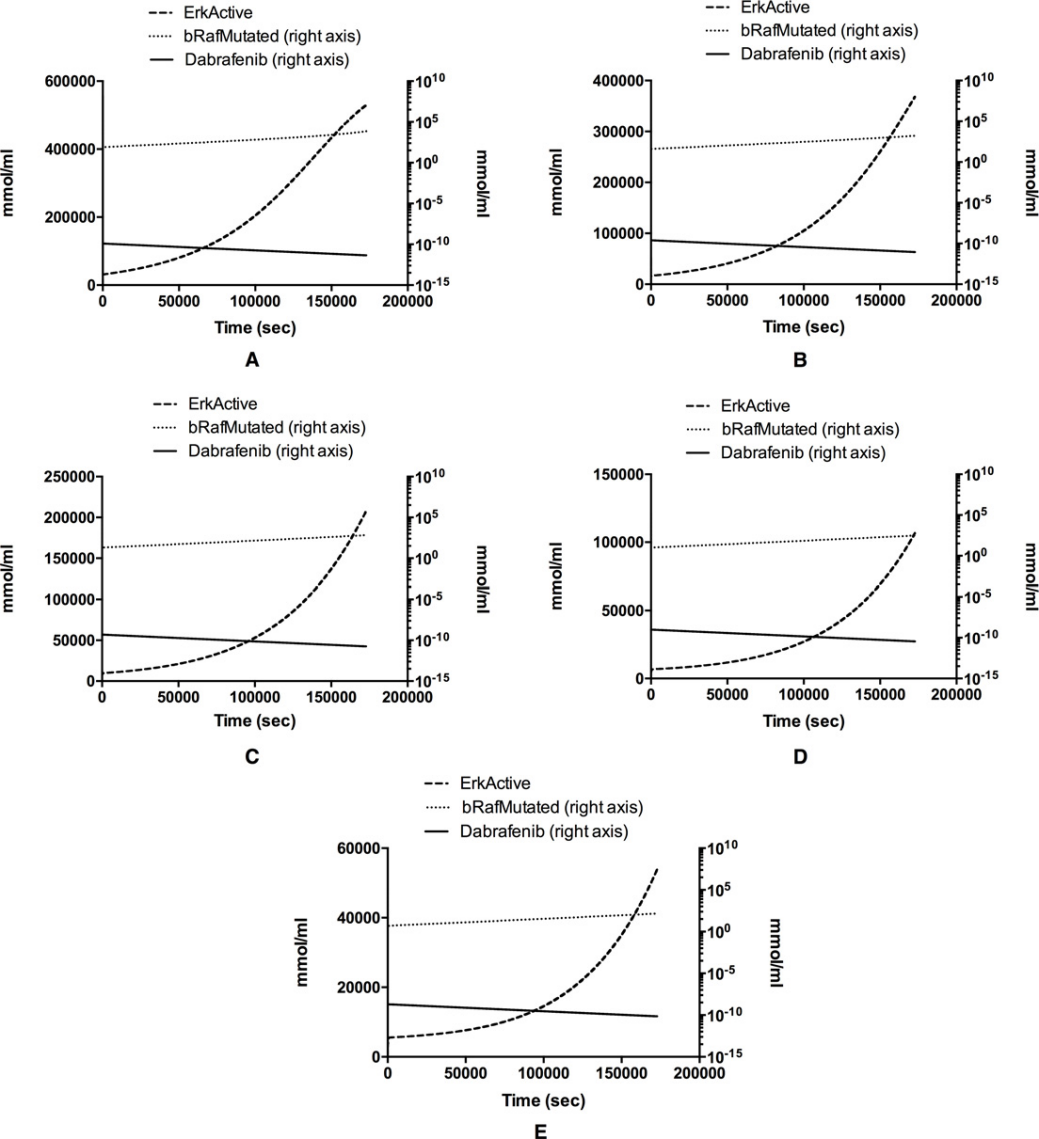
pERK, B-RAF and Dabrafenib in silico dynamics. pERK concentrations decrease during Dabrafenib treatment in A375 melanoma cell line. The behavior of pERK is directly correlated to the Dabrafenib concentration. Different dosages of Dabrafenib are shown (1nM = 1e-9 mmol/ml): 1.25e-10 mmol/ml (A), 2.5e-10 mmol/ml (B), 5e-10 mmol/ml (C), 1e-9 mmol/ml (D) and 2e-9 mmol/ml (E). In all the panels the right y-axis dotted lines represent bRafMutated concentrations while solid lines represent Dabrafenib concentrations.

The dynamics of both pERK and B-RAF mutated were modeled (Fig 4). ERK in silico concentration was set to 600000 mmol/ml. Five panels are shown. Each panel depicts the pERK, B-RAF mutated and Dabrafenib dynamics in 48 hours of simulation at different Dabrafenib dosage i.e., 0.125 nM (A), 0.250 nM (B), 0.500 nM (C), 1.0 nM (D) and 2.0 nM (E). When no treatment is administered (DMSO) pERK concentration reached 571950 mmol/ml at time 48 h. With different dosages of Dabrafenib, pERK concentrations reached 529602 mmol/ml (A), 368352 mmol/ml (B), 207518 mmol/ml (C), 106758 mmol/ml (D) and 53385 mmol/ml (E), showing, respectively, the percentage of inhibition reported in Table 1.

The reduction of pERK concentrations is in agreement with that observed in vitro.

**Fig 5:**
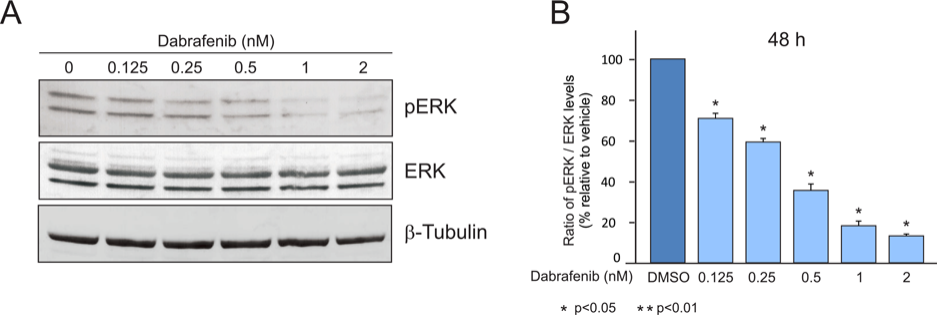
Western blot analysis of p-Erk and total ERK in A375 melanoma cell line after treatment with different doses of Dabrafenib for 48 hours (A). p-ERK signal was normalized with total ERK signal (B), SD and means of the normalized p-ERK values were reported.

Fig 5 highlights western blotting plots. Also in this case, we obtained a good agreement with the in silico results. In conclusion, both from the model results and from the in vivo experiments, we can observe that p-ERK levels drop down due to inhibitor activity of Dabrafenib over B-RAFV600E protein. We analyzed p-ERK as it is one of the key protein kinase involved in cell proliferation signaling. An important aspect related to the inhibition of B-RAFV600E protein is that a small fraction of treated melanoma patients develops resistance mechanisms that make the therapy not more effective. The model can be useful to analyze the complex PI3K/AKT and MAPK pathways in order to discover proteins that may cause these resistance phenomena.

## Conclusions

We presented a computational model that simulates both PI3K/AKT and MAPK pathways and their interactions, in order to analyze the cascade reactions responsible for melanoma development. We simulated a therapy intervention i.e., the administration of a well-known B-RAF inhibitor, Dabrafenib. This study has shown how computational models can be useful tools for investigating and comparing the biological behavior of signal transduction path- ways as they can suggest new hypotheses to explain the observed biological data and help understand the dynamics of how the pathway functions. Furthermore, computational models can be readily used to investigate different disease states and suggest how drug treatment could be improved to better combat the effects of the disease. We believe that our model is a good representation of the PI3K/AKT and MAPK with activated ERK pathway which can be expanded and applied in the future to further investigate the dynamics of resistant mechanisms in order to suggest new intervention to suggest new therapeutical interventions.

## Supporting Information

S1 Archive In the zip archive, there are three different available versions of the model: File PI3K_AKT_Final_V2.1.cps, physiological model i.e., no B-RAF mutation; File PI3K_AKT_Final_V2.1_A375.cps, A375 cell line model i.e, with B-RAF mutation; File PI3K_AKT_Final_V2.1_A375_Dabrafenib.cps, complete model with both B-RAF mutation and Dabrafenib inhibitor set at the lower dosage. (ZIP)

## References

[1] McCubrey JA, Steelman LS, Chappell WH, Abrams SL, Franklin RA, Montalto G, et al Ras/Raf/MEK/ERK and PI3K/PTEN/Akt/mTOR Cascade Inhibitors: How Mutations Can Result in Therapy Resistance and How to Overcome Resistance. Oncotarget. 2012;3: 1068–1111. 23085539 10.18632/oncotarget.659 PMC3717945

[2] Libra M, Malaponte G, Navolanic PM, Gangemi P, Bevelacqua V, Proietti L, et al Analysis of BRAF Mutation in Primary and Metastatic Melanoma. Cell Cycle. 2005;4: 1382–1384. 16096377 10.4161/cc.4.10.2026

[3] Miller AJ. Mihm MC. Melanoma. N Engl J Med. 2006;355: 51–65. 16822996 10.1056/NEJMra052166

[4] Wolchok JD, Saenger YM. Current topics in melanoma. Curr Opin Oncol. 2007;19: 116–120. 17272983 10.1097/CCO.0b013e32801497c6

[5] Garnett MJ, Marais R. Guilty as charged: B-RAF is a human oncogene. Cancer Cell. 2004;6: 313–319. 15488754 10.1016/j.ccr.2004.09.022

[6] Ascierto PA, Kirkwood JM, Grob JJ, Simeone E, Grimaldi AM, Maio M, et al The role of BRAF V600 mutation in melanoma. J Transl Med. 2012;10: 85 10.1186/1479-5876-10-85 22554099 PMC3391993

[7] Tsao H, Goel V, Wu H, Yang G, Haluska FG. Genetic interaction between NRAS and BRAF mutations and PTEN/MMAC1 inactivation in melanoma. J Invest Dermatol. 2004;122: 337–34. 15009714 10.1046/j.0022-202X.2004.22243.x PMC2586668

[8] Perkinton MS, Ip J, Wood GL, Crossthwaite AJ, Williams RJ. Phosphatidylinositol 3-kinase is a central mediator of NMDA receptor signalling to MAP kinase (Erk1/2), Akt/PKB and CREB in striatal neurones. J Neurochem. 2002;80: 239–254. 11902114 10.1046/j.0022-3042.2001.00699.x

[9] York RD, Molliver DC, Grewal SS, Stenberg PE, McCleskey EW, Stork PJS. Role of phosphoinositide 3-kinase and endocytosis in nerve growth factor-induced extracellular signal-regulated kinase activation via Ras and Rap1. Mol Cell Biol. 2000;20: 8069–8083. 11027277 10.1128/mcb.20.21.8069-8083.2000 PMC86417

[10] Zhuang ZY, Xu H, Clapham DE, Ji RR. Phosphatidylinositol 3-kinase activates ERK in primary sensory neurons and mediates inflammatory heat hyperalgesia through TRPV1 sensitization. J Neurosc. 2004;24: 8300–8309. 10.1523/JNEUROSCI.2893-04.2004 PMC6729698 15385613

[11] Aksamitiene E, Kiyatkin A, Kholodenko BN. Crosstalk between mitogenic Ras/MAPK and survival PI3K/AKT pathways: a fine balance. Biochemical Society Transactions. 2012;40: 139–146. 10.1042/BST20110609 22260680

[12] Castiglione F, Pappalardo F, Bianca C, Russo G, Motta S. Modeling biology spanning different scales: An open challenge BioMed Research International, 2014, 10.1155/2014/902545 PMC4124842 25143952

[13] Gullo F, van der Garde M, Russo G, Pennisi M, Motta S, Pappalardo F, et al Computational modeling of the expansion of human cord blood CD133+ hematopoietic stem/progenitor cells with different cytokine combinations. Bioinformatics, 2015;31: 2514–2522. 10.1093/bioinformatics/btv172 25810433

[14] Pappalardo F, Palladini A, Pennisi M, Castiglione F, Motta S. Mathematical and computational models in tumor immunology. Mathematical Modelling of Natural Phenomena. 2012;7: 186–203.

[15] Vennepureddy A, Thumallapallya N, Nehrua VM, Atallahb JP, Terjanianb T. Drugs and Combination Therapies for the Treatment of Metastatic Melanoma. Journal of Clinical Medicine Research. 2016;8: 63–75. 10.14740/jocmr2424w 26767073 PMC4701060

[16] Brown KS, Hill CC, Calero GA, Myers CR, Lee KH, Sethna JP et al The statistical mechanics of complex signaling networks: nerve growth factor signaling. Phys Biol. 2004;1: 184–195. 16204838 10.1088/1478-3967/1/3/006

[17] Hoops S, Sahle S, Gauges R, Lee C, Pahle J, Simus N, et al COPASI—a COmplex PAthway Simulator. Bioinformatics. 2006;22: 3067–3074. 17032683 10.1093/bioinformatics/btl485

[18] Kanehisa M, Goto S, Kawashima S, Okuno Y, Hattori M. The KEGG resource for deciphering the genome. Nucleic Acids Res. 2004;32: 277–80. 10.1093/nar/gkh063 PMC308797 14681412

[19] Howe EA, Sinha R, Schlauch D, Quackenbush J. RNA-Seq analysis in MeV. Bioinformatics. 2011;27: 3209–10. 10.1093/bioinformatics/btr490 21976420 PMC3208390

[20] Bajzer Z, Strehler EE. About and beyond the Henri-Michaelis-Menten rate equation for single-substrate enzyme kinetics. Biochem Biophys Res Commun. 2011;417: 982–985. 10.1016/j.bbrc.2011.12.051 22206668

