# Supplementary material for: Computational Modeling of PI3K/AKT and MAPK Signaling Pathways in Melanoma Cancer
Source: PLoS One. 2016 Mar 25;11(3):e0152104. doi: 10.1371/journal.pone.0152104 (PMC4807832; doi:10.1371/journal.pone.0152104)
Supplement: S1 Archive — (ZIP) [file pone.0152104.s001.zip › Source_Models/Explanation_Source_Models.docx]

The model requires COPASI to run, that is freely available following this [link](http://copasi.org/).

In the zip archive, there are three different available versions of the model:

- File ***PI3K_AKT_Final_V2.1.cps***, physiological model i.e., no B-RAF mutation.
- File ***PI3K_AKT_Final_V2.1_A375.cps***, A375 cell line model i.e, with B-RAF mutation.
- File ***PI3K_AKT_Final_V2.1_A375_Dabrafenib.cps***, complete model with both B-RAF mutation and Dabrafenib inhibitor set at the lower dosage. Click [here](http://www.francescopappalardo.net/Bioinformatics_PI3K_AKT_Model/PI3K_AKT_Final_V2.1_A375_Dabrafenib.cps.zip) to download the source code of the model.
